# Editors between Support and Control by the Digital Infrastructure — Tracing the Peer Review Process with Data from an Editorial Management System

**DOI:** 10.3389/frma.2021.747562

**Published:** 2021-10-19

**Authors:** Judith Hartstein, Clemens Blümel

**Affiliations:** ^1^ German Centre for Higher Education Research and Science Studies (DZHW), Berlin, Germany; ^2^ Department of Social Sciences, Faculty of Humanities and Social Sciences, Humboldt-Universität zu Berlin, Berlin, Germany

**Keywords:** editorial management systems, peer review, process generated data, digital transformation of scholarly publishing, digital infrastructure

## Abstract

Many journals now rely on editorial management systems, which are supposed to support the administration and decision making of editors, while aiming at making the process of communication faster and more transparent to both reviewers and authors. Yet, little is known about how these infrastructures support, stabilize, transform or change existing editorial practices. Research suggests that editorial management systems as digital infrastructures are adapted to the local needs at scholarly journals and reflect main realms of activities. Recently, it has been established that in a minimal case, the peer review process is comprised of postulation, consultation, decision and administration. By exploring process generated data from a publisher’s editorial management system, we investigate the ways by which the digital infrastructure is used and how it represents the different realms of the process of peer review. How does the infrastructure support, strengthen or restrain editorial agency for administrating the process? In our study, we investigate editorial processes and practices with their data traces captured by an editorial management system. We do so by making use of the internal representation of manuscript life cycles from submission to decision for 14,000 manuscripts submitted to a biomedical publisher. Reconstructing the processes applying social network analysis, we found that the individual steps in the process have no strict order, other than could be expected with regard to the software patent. However, patterns can be observed, as to which stages manuscripts are most likely to go through in an ordered fashion. We also found the different realms of the peer review process represented in the system, some events, however, indicate that the infrastructure offers more control and observation of the peer review process, thereby strengthening the editorial role in the governance of peer review while at the same time the infrastructure oversees the editors’ performance.

## Introduction

In the last 15 years, novel digital infrastructures of different forms and shapes have been established, aiming at supporting communication, dissemination and evaluation of scientific research (Van Noorden, 2014; Taubert, 2016; Blümel, 2021). Though many would agree that novel practices relating to different platforms have emerged (such as, for example, social bookmarking sites), many open questions remain as to whether such infrastructures have profoundly changed existing processes, values or practices of knowledge production (Horbach and Halffman, 2019). One of the core areas witnessing the introduction of digital tools is the realm of scientific publishing and peer review in particular (Jubb, 2015, pp.16). Many journals now rely on editorial management systems (Taubert, 2012), which are supposed to support the administration and decision making of editors, while aiming at making the process of communication faster and more transparent to both reviewers and authors (Mendonça, 2017). Editorial management systems are digital infrastructures processing the submission, evaluation and administration of scholarly articles. According to Mendonça (2017), they are “designed to perform the management of manuscripts from submission to final decision, offering greater control, automation and logging of processes that were once manually done”.

The institution of scholarly peer review as the main instance for scientific quality assurance appears to be comparably stable since more than three hundred years, despite several technical changes (Reinhart, 2010; Pontille and Torny, 2015; Horbach and Halffman, 2019). The idea to apply peer culture to science in order to protect the community of knowledge makers emerged in the Royal Society in late 17th century (Shapin 1994). Yet, as Horbach and Halffmann (2019) have outlined, peer review as an institutional practice at scholarly journals has a far more recent history, beginning in late 19th century in scientific societies which established the first disciplinary scholarly journals (Csiszar, 2018). Since then the success of peer review in science was unprecedented and can be seen in the various ways peer review has been integrated for the evaluation of scholarly output, with varying expectations as to what it is to accomplish. Today, peer review is not only practiced to judge the quality and appropriateness of scholarly manuscripts for specific journals, but also to evaluate grant proposals (Reinhart, 2010), persons (such as in calling committees) (Kleimann and Hückstädt, 2021) or even research organizations (Röbbecke and Simon, 1999). While there are similarities between the different ways of using peer review, peer review for manuscript evaluation is specific in the way it is embedded within the organization of scholarly journals (Hirschauer 2004). Scholarly journals invest considerable effort in maintaining peer culture by establishing close links to authors, reviewers, and (guest) editors (Weller, 2001). Hence, peer review processes at scholarly journals can be perceived as community work with the aim to establish consistent and sustainable networks between all actors involved.

Peer review at scholarly journals, however, does also have a function in protecting scientific autonomy by safeguarding quality. According to Guston (2001), there is a social contract granting autonomy and self-regulation to science only if scientific quality and productivity is ensured. There is much consensus about peer review for manuscripts being a major instrument for quality control despite differences what that means in practice (Campanario, 1998a; Campanario, 1998b). Yet, calls for reforms in scholarly peer review have grown louder particularly emerging from critics about biases in peer review (Cicchetti et al., 1992; Harnad, 1983; Bornmann 2005). Such critics also fueled debates about new forms of open peer review, as technological or organizational innovations are imagined to ultimately alter editorial practices at scholarly journals (Ross-Hellauer et al., 2017). It appears that some of these calls presuppose knowledge about the complex interplay of actors and technologies in editorial processes. Yet, despite much research about biases in peer review, little do we know about the actual processes of peer review, and even less so about new practices and technologies supporting peer review (Jubb, 2015, p.13). While different studies about the roles and tasks of both reviewers and editors were published (Hirschauer, 2010; Glonti et al., 2019), editorial practices are only rarely investigated (Weller, 2001). That is why it would be difficult to make claims about changes between a pre-digital and a digital scholarly journal world: we simply do not know enough about organizational practices of peer review as such, though research about peer review has grown recently (Batagelj et al., 2017). However, digital infrastructures supporting peer review have been established to support decision making and communication in the process of publishing scholarly manuscripts (Horbach and Halffman, 2019), enabling the investigation of the corresponding new digital practices.

Currently there is so far no systematic analysis of the structure of practices in the peer review process. Many researchers, reviewers and editors do have opinions about the roles and responsibilities of both editors and reviewers (Glonti et al., 2019), some of which contradict each other (Glonti et al., 2019, p.1). Moreover, the characteristics of both reviewers and editors are explored to a significant extent (Hirschauer, 2010, 73). Yet, little is actually known about how the peer review process is practiced and how it is supported through administrative procedures, such as how reviewers are invited (Bös, 1998), how reviews are maintained, or decisions are communicated; activities which might be considered administrative in the first place. Though many agree that scholarly publishing and peer review are social processes (Reinhart, 2010), investigations about the processes of scholarly publishing and peer review are rare, given that persons engaged in these processes “actively resist investigation” (Hirschauer, 2010, 73). What is more, scholarship about peer review lacks from a structural perspective on that process, e.g., how much time and resources are bound by which kind of activities in the process of handling manuscripts at scholarly journals. To the best of our knowledge, our analysis is one of the very few quantitative analyses of these processes.

Against that background, the goals of this research are 1) to explore the structure of activities in the process of handling manuscripts based on insights gained from process generated data from an editorial management system, taking Schendzielorz’s and Reinhart’s (2020) model of the peer review process as a conceptual heuristic. Secondly 2), we intent to gain insights into the ways editorial management systems shape or transform editorial practices, i.e., to explore the ways of how the technology has been implemented in the journal. We do this by comparing the model laid out in the patent for the infrastructure (Plotkin, 2009) with the empirical data generated by the infrastructure. Recent research into platforms (Blümel, 2021) has argued that novel digital infrastructures are considered as agents of change for scholarly practices by incorporating several functions relevant for decision making and quality control. More specifically, we hence thirdly 3), also aim at exploring as to whether one can find traces of automated decision making, something which could more radically alter editorial peer review and scholarly publishing. Our goal in posing these questions is to gain insights into how novel editorial management systems change or stabilize knowledge production.

Empirically, we use digital traces from an editorial management system in order to gain insights into how the digitalized peer review process looks like. It has been stated that such infrastructures are also a source for negotiating innovations in peer review, as “the system plays a major role in connecting and coordinating the various editorial practices” (Horbach and Halffman, 2020, p.11). Exploring data from that infrastructure, we complement others’ research investigating views and perceptions of peer review practices with a new procedural perspective explicitly taking algorithms and digital affordances of digital infrastructures into account. A closer look at process generated data allows us to explore which elements of the peer review and decision making process in scholarly journals are communicated and shared on a digital infrastructure, how the process of peer review is transformed into countable events and made visible.

We focus our analysis on editorial peer review, that is, processes related to editorial selection, management and decision making. Editors are often perceived as the gate keepers of science (Crane, 1967), distributing credit and reputation by deciding about papers to be published against field and journal specific values and criteria (Jubb, 2015, p.14). But, as Schendzielorz and Reinhart (2020) recently have pointed out, editorial work can also partly be considered as administrative, “taking into account that peer review takes place in an organizational setting” (ibid., p.18). Administrative work at journals then comprises, for instance, the “handling and coordination of manuscripts” (ibid.). Whether digital infrastructures such as editorial management systems are transforming the peer review process with regard to these two tasks is hard to tell, given the difficulties of exploring the process. Some authors claim transformative changes would be at play for practices of editors handling manuscripts: Taubert (2012) for instance has stated that journal editorial management systems standardise the peer review process and constrain the degrees of freedom for editors. On the other hand, it has been argued that editorial management systems support the editorial role and reproduce or may even increase the instruments to regulate, administrate and ultimately control the process (Mendonca, 2017). One of the reasons for the rising significance of editorial practices is the increase of self-control of scholarly journals emerging from the digital transformation of the process induced by the editorial management system. Administrative practices of coordinating manuscripts, selecting reviewers and managing consultations are increasingly difficult to separate from observational practices without direct effect on the process, which can be, according to Schendzielorz and Reinhart (2020, p.19), considered as relevant for controlling the peer review process. Some of these activities, formerly external to the normal administrative editorial work, may now be automated by the infrastructure, leading to novel control technologies which may also put the editorial role under stronger pressure. For instance, the editor might become aware of their own velocity in deciding or transferring manuscripts (Mrowinski et al., 2016), hence administrating the process. Such claims are difficult to make given the limitations many studies on editorial peer review face. However, based on our analysis, we explore what can be known from editorial management systems and in what ways decisions jointly emerge from editorial decision and structures provided by the infrastructure. These changes in the ways of how the infrastructure is used may alter the boundaries between different types of practices carried out within organizations handling peer review (see next theoretical section), and ultimately the editorial role as such.

Although editorial management systems have been introduced in the dawn of the current millenium, research about process generated data from these systems within scholarly journals has − to the best of our knowledge − not been published so far. Our approach therefore is explorative; we aim at making these data accessible and provide early interpretations of their structures. The main aims of our study are hence the following: By investigating process generated data from a publisher’s editorial management system, we aim to explore the ways by which the digital infrastructure is used and how it represents the process of peer review. How does the infrastructure support, strengthen or restrain the editors’ agency for administrating the process? The original ideas and values attached to the system are expressed well by the developers of the technology, who, by aiming at facilitating the process of peer review, defined major entities and activities for administrating manuscripts. Plotkin (2009) – in laying out the basis of the editorial management system used in our case – patented a “process for computer implemented manuscript review” and described a prototypical journal peer review process. The patent depicts peer review as an ordered process with actions (such as sub-processes, documents and stored data) and bifurcations (see Figure 3). Sometimes, it is mentioned, who is involved in the said actions, but sometimes not. The focus of the patent is on how to facilitate the peer review process in a digital infrastructure. We aim to compare empirical process generated data with this idealized process provided with the patent, because the processual data reflect local adaptations and uses of these technologies emerging from concrete demands of authors, reviewers and editors in the configurations of a journal (Horbach and Halffman, 2019, p.2), but are at the same time also constrained by the initial definition of roles and processes set up by the developers of the technology (Krüger et al., 2021). While these technical adaptations reflect the processual or organizational demands, they may also create novel arenas for monitoring and control neither foreseen by the developers nor by organizational professionals of peer review work. In order to make such comparisons, we employed social network analysis with the events in the manuscript lifecycle as nodes which are connected through their relation in time.

Our contribution is organized as follows. In the next section, we introduce the theoretical framework and main perspectives. In the third section, the data and their preparation are described in more detail, elaborating on why a social network approach appears to be suitable for exploring relationships between events of the editorial process mediated by the system. We then continue by presenting major outcomes of the study, followed by a discussion about the editorial processes mediated by editorial management systems, and the role of automated decision making.

## Theoretical Considerations

In this work, editorial management systems are perceived as an infrastructure supporting peer reviewed scientific publishing. Hence, we draw from a growing theoretical literature on digital infrastructures from science and technology studies and also from literature about processes and practices in peer review from the social studies of science.

Editorial management systems are perceived as an infrastructure in this work. According to Star and Bowker, infrastructures are used to enable, maintain and control collaboration among different actors (Star, 1999; Star and Bowker, 2006). Moreover, infrastructures can be seen as structures emerging from “situated knowledges”, a term coined by Haraway (1988) with regard to people and communities with partial perspectives. Also, with Friedman and Nissenbaum (1996), we argue, that the infrastructure itself is shaped by assumptions from its developers about how the world is like and should be. Also, infrastructures in science such as editorial management systems are embedded in highly structured practices, such as the selection of reviewers, formats for presenting and evaluating manuscripts from which they cannot be separated. Such heterogeneous uses influence and transform the infrastructure as an assemblage of situated digitally mediated practices (Horbach and Halffman, 2020, p.2), that is, practices which can only be understood in the context of their local usage (e.g., a specific function accomplished within the context of a specific journal). The use of editorial management systems as digital infrastructures for the management of collaboration hence requires processual knowledge about the peer review process. Consequently, infrastructures may best be understood as manifestations of specific operations or sometimes even of a whole process (Niewöhner, 2014, 6).

Editorial management systems may then be interpreted as representations and manifestations of the peer review process which is itself an internal element of the self-governance within the sciences. Recently Schendzielorz and Reinhart (2020) provided a scheme for analysis of peer review with special regard to its control function in a decision-making process for the distribution of scarce resources. While they draw in their examples from grant peer review, they explicitly claim their depiction to enable comparative analyses of different peer review processes along the elements of a minimal process: postulation, consultation, decision and administration. At the same time, they emphasize a power perspective with regard to different degrees of involvement for actors, their role and participant status. They point out that taking into account different regimes of power in peer review processes as government requires exploring how interests are transformed into processes, that is, sequences of events and formalized activities (ibid., p.23). While the elements provided are not always easy to distinguish empirically, it appears plausible to assume that they may reflect different roles in that process. Editorial management systems may be understood as aiming at representing such abstract roles and processual elements.

At the same time, however, editorial management systems as digital infrastructures transform that process by defining sequences, ends, values and evaluation criteria, which are inscribed already in the production process of such devices (see Krüger et al., 2021). These values and criteria can, for instance, be captured by studying aims and means of the patent (Plotkin, 2009) which serves as the technological basis for the editorial management system from our investigation. In this principal depiction, the digital infrastructure of the editorial management system is presented to foster values such as timeliness and comprehensiveness. Moreover, acceleration, control and efficiency have been main arguments for establishing editorial management systems in the first place (Jubb, 2015; Mendonça, 2017), putting pressure on publishers and editors of journals to implement streamlined procedures.

## Materials and Methods

In this paper, we present an empirical case study: processual data from a journal management system provide insights into how the peer review process is carried out at four journals of a specific publisher in the biomedical field. Since we draw from data of one publisher, we cannot make systematic claims about the usage of editorial management systems, but rather intend to generate new questions and perspectives for research in this area. In the data used for our investigation, we see traces of actions and participant roles in different processes. The actions are attributed with manuscripts they belong to, and points in time when they were carried out, which is why we are able to infer the order of actions, choices at forks and pace of the process.

The publisher provided us with processual data from their journal management system during an earlier research project with a focus on evaluation practices and sources of biases in peer review. The publisher uses the system EJournalPress to manage their editorial peer review lead by full-time staff editors in a shared office space. They employ single-blind peer review, which means that the reviewers are aware of the authors’ identities unless otherwise requested by the authors. Also, the review-process is partly made transparent ex-post, expressed by the fact that published papers are accompanied by online supplementary material comprised of the reviewers’ comments, editorial decision letters and communication between authors and editorial office, unless otherwise requested by the authors. As acquiring complete inventory data from not fully open peer review is very difficult, we used the hereby presented study to exploit more of the potential of the data.

We use the perspective of the infrastructure by studying the recorded events it has created as a result of actions by different actors. The infrastructure models the peer review process along the way of submitted (versions of) manuscripts, which enter the system during submission and pass through different stages afterwards. For some time, the manuscript items are actively maintained when they undergo consultation eventually, when they are decided about, and when the editorial decision is communicated to the authors and/or the manuscript is sent to production. When the process is finished, the manuscript lies dormant in the database. Following an ethnographic approach to infrastructures, we reconstruct sequences of the stages passed by the manuscript, taking into account how long it takes for manuscripts to pass from one stage to another. It is not our goal, however, to make a life cycle analysis of manuscripts at this publisher. Rather, we intend to infer editorial practices from these sequences which may jointly emerge from the editors’ actions and the infrastructure, being aware that our perspective is limited.

Different to what the patent for the technology suggests, the actual use of the infrastructure may be particularly complex, revealing the difficulties in managing and maintaining collaboration among different types of actors. While we do not have empirical material about the interpretations of the process by the actors themselves, processual data and the sequences of events may at least allow for abductive reasoning about how the editorial role is structured, and, in light of the literature about peer review, transformed, by using the infrastructure.

Exploring a digital infrastructure without actually having access to it is challenging. This to be acknowledged, Seaver (2017) described some “tactics for the ethnography of algorithmic systems”, of which we take up the tactic of scavenging in our work: using the pieces of information accessible to us while at the same time keeping in mind that we only see a part of the whole picture. Christin (2020) coined the term *algorithmic refraction* aiming at “bypassing algorithmic opacity” to address drawing conclusions under the circumstances of incomplete information. The latter means to us that while the system itself is hidden from us, we use what we have access to: traces of how the digital infrastructure is used. Thus, we bypass the (to us) opaque system, but can nevertheless infer insights about the practices and implementations of the peer review process in question.

Nevertheless, our approach leads to methodological questions of digital inquiries. Marres (2017) points out that by dealing with data from digital infrastructures, research agency is twisted: the data often prompt the researcher to their perspective and methodology, resulting in that “digital research requires an at once critical and creative approach to method” (p.115). Given that our data set is situated and that digital practices are related and aligned by the infrastructure, we follow the infrastructures and aim at studying how they structure and reflect the practices of its users. Our results may inform future studies and allow for making more detailed observations of the editorial process.

Hereinafter, to demarcate different perspectives, we speak of *actions* or *activities*, when we refer to what is *done*, and we talk about *events* or *stages*, when we refer to what is *recorded* in the infrastructure and found in the data traces. An example would be a researcher filling in a form in a web frontend including uploading a manuscript (activity/action), which the infrastructure would be recording as “Manuscript submitted by user X” (event/stage). In the database entry, we would later discover this as a digital trace of the action performed. In other words, events can be thought of as the ways of how activities are conceived by the infrastructure. Also, when we conceptually refer to the process, we write *element* or *component* for *conglomerates of either actions or events* which belong together.

### Data

The data stem from the editorial management system eJournalPress and the focal data used here are the “history”-information of 14,392 manuscript files referring to 17,109 manuscript versions processed in the years 2011 and 2015 in the infrastructure for four of the publisher’s journals, which depict the manuscript life cycle from the infrastructure’s point of view. This data represents a full inventory of manuscript version histories for the given years and journals, covering all submitted manuscripts whether published in the end, or not. For our analyses, only the internal representation of the process in the systems database was used, we did not investigate the frontend of the editorial management software. Also, the database is, of course, more complex and stores lots of information from user accounts to e-mail communication, but our analyses refer exclusively to the manuscript life cycle.

The raw manuscript histories were parsed from xml-files to a table and are rather simple in structure, but lack a documentation. We found multiple observations for each manuscript with a stage name, a time stamp and two pseudonymized person-identity numbers (hereinafter, person-IDs), in the system originally identifying individual users assigned to it – the person who triggered an event and the person affected by an event (judging by the xml-tags assigned to the information). In total, 278,098 events were filed in the database. The event information was further enriched with year of submission, pseudonym of journal, and by (pseudonymized) data about the roles (editor, author, reviewer or none) of the person-IDs with regard to the respective manuscripts. The description of the variables was mainly derived from the field names, their values and the xml-structure in the raw data and is given in Table 1.

**TABLE 1:** Description of variables.

Variable name	Variable description
Key	Manuscript identifier with version indicator
stage.name	Name of the event that happened
stage.triggered.by.person.id	Person acting
stage.affective.person.id	Person affected
journal_pseudo	The journal submitted to
journal_year	The year submitted in
stage.triggered.by.role	Role of person acting (relative to manuscript)
stage.affective.role	Role of person affected

**FIGURE 1:**
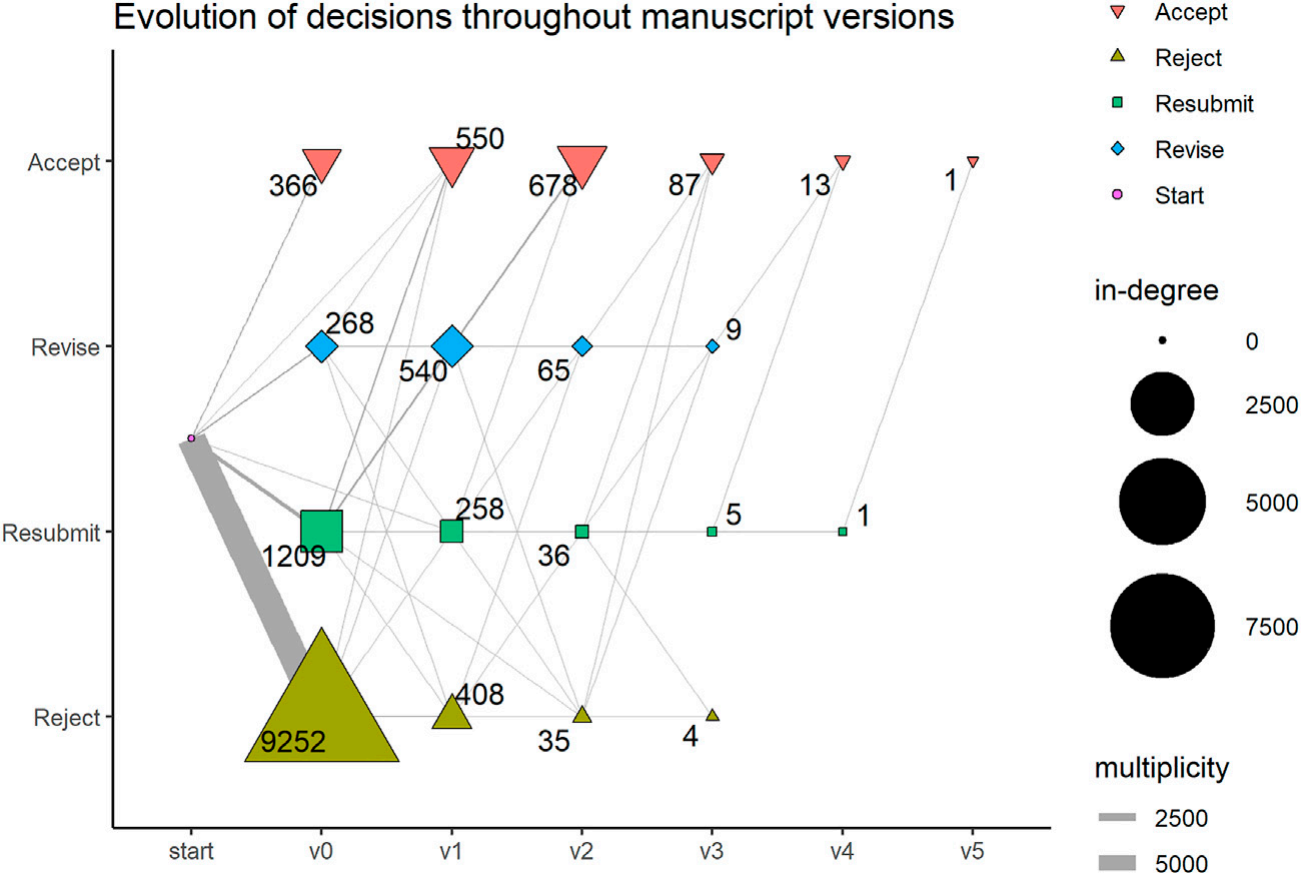
The figure shows the decisions for the original manuscript version (v0) and resubmitted versions (v1–v5). The numbers indicate, how often a specific decision is reached for the respective version (the in-degree of the node). The edge widths show, how many manuscripts experience the respective evolutionary path.

For the investigation of actions with regard to the different roles in the process, the whole dataset was used. However, we decided to restrict our analysis of the sequence of stages to the 14,391 first-version manuscripts with 206,896 events to avoid obfuscation of the prototypical process by manuscript versions with a past. (For one manuscript, no first version was present in the inventory – hence, the difference between 14,392 and 14,391 manuscripts). As was said earlier, the infrastructure understands the process along the stages, a manuscript version passes through. This means that a manuscript will usually loop through the review process more than once, depending on the editorial decision–in our case up to six times. A pre-screening of our data showed that the first round of peer review differs from the subsequent ones. Of all 11,103 manuscripts which make it to a decision at least in one round, the first submitted version is rejected in the vast majority of the cases, whereas manuscripts which make it through the first round, stand a good chance to be accepted in the later stages, as is shown in Figure 1. That means, the first round is crucial to the manuscript’s fate and, moreover, the preceding rounds might predetermine the shape of the process in the later rounds. That is why we also focus our structural analysis of the peer review process on this first round of peer review.

### Methods

The editorial peer review process for a single manuscript version is investigated from three perspectives: the perspective which considers the sequencialization (which stages are passed in which order) of the process, the pace (how long does a step take) of the manuscript during the process and the magnitude (how many manuscripts go along a specific path). For this purpose, we use network analysis: the vertices represent the stages and a (directed) edge is drawn from one stage to another when it is directly following in one item’s history. Additionally, source and target vertices were inserted to make start and end of the process visible in plots. The complete network is comprised of 72 vertices and 221,287 edges.

**FIGURE 2:**
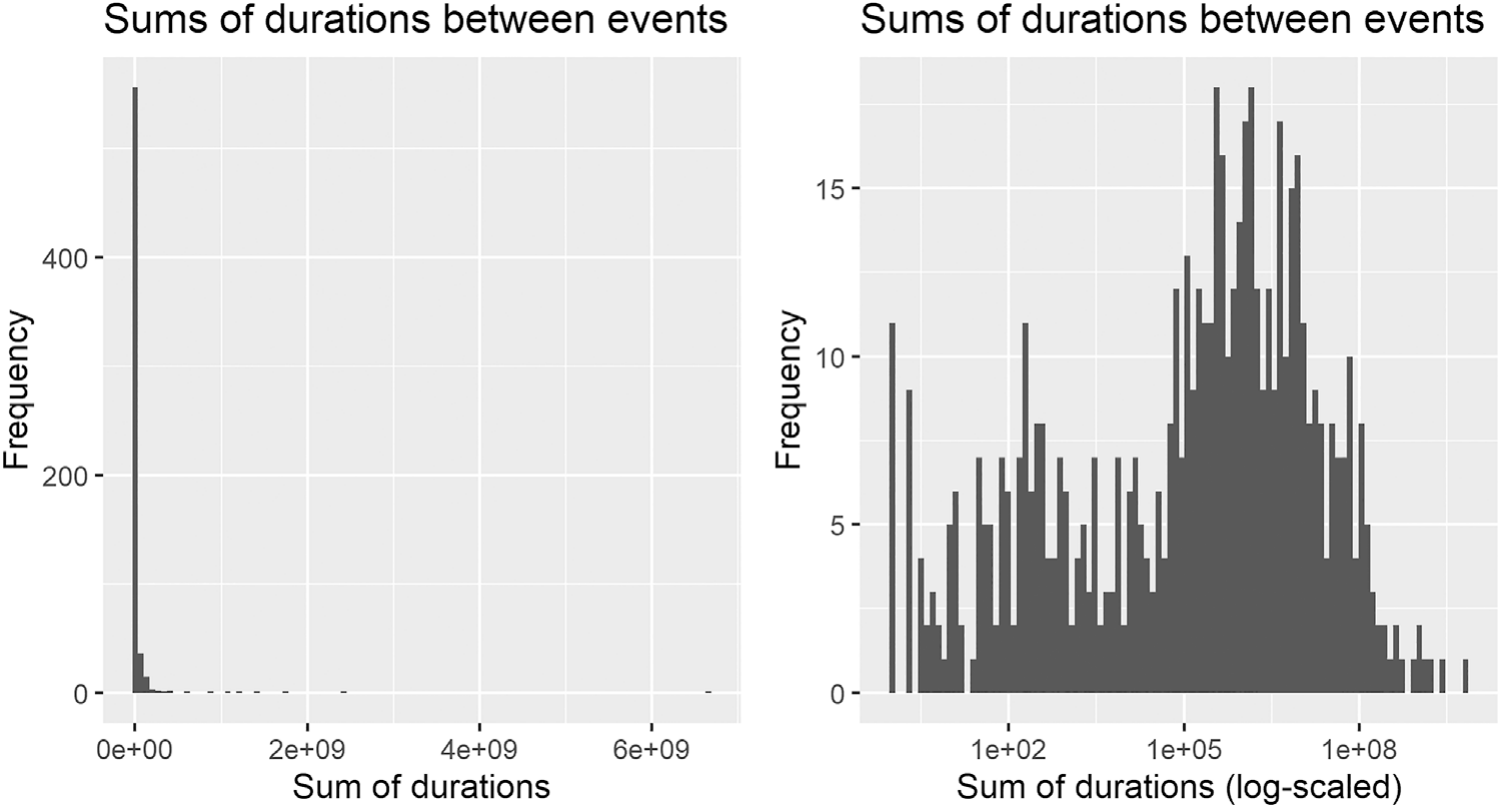
Histograms of sums of durations between successive events in the process: The distribution is skew to the left; the log-scaled distribution is better leveled (Remark: 14 durations of length 0 are left out in the logarithmized plot).

For most of the analyses, a simplified network was used: loops were removed and multiple edges between the same two vertices were reduced to one. This led to a network of 623 edges with a density of d = 0.12. The edges carry two attributes: the multiplicity (how often two events occur in direct sequence in the items histories) and, as weight attribute for layout algorithms, the logarithm of the sum of durations between two vertices. The logarithm was chosen because the time between stages is distributed skew to the left (see Figure 2). This is partly caused by several automated steps present in the process, which can take only one second to happen. The network was then investigated iteratively, each descriptive step pointing to a new direction to follow and the insights gained were grouped together and will be discussed against each other in the end. The quantitative analyses were performed with the use of R (R Core Team, 2020) and the following contributed packages: igraph (Csardi and Nepusz, 2006), tidyverse (Wickham et al., 2019), lubridate (Grolemund and Wickham, 2011), data.table (Dowle and Srinivasan, 2021) and ggraph (Pedersen, 2021).

## Results

Drawing from the theoretical considerations explained above, we first present results regarding the different roles which the editorial management system supports and enables in order to understand how the governance of the process is represented and performed by the editorial management system. The analysis may also provide first insights to what extent the events recorded are automatically generated. In the second section of the results, we aim at tracing the order of the events in the editorial management system.

### Roles in the Peer Review Process

As Schendzielorz and Reinhart (2020) have outlined, differences in the governance of peer review systems become visible not only in how the process of peer review is transformed in a sequence of events, but also in how the different actors take part in this process and how they affect each other’s actions. These different forms of actors can be best perceived as specified roles, describing and demarcating specific types of activity, that is, for instance, making claims (authors), handling and coordinating manuscripts (editors), evaluating claims (reviewers) and deciding about whether to publish a manuscript or not (editors).

The editorial management system makes these different roles visible, by attributing person-IDs as authors, editors and reviewers to manuscripts. Additionally, actions were recorded for person-IDs not having a role assigned for the respective manuscript. The biggest share – 112,475 out of all 278,098 events filed in the database – were triggered by editors, or, to be more precise, by actors assigned an editorial role for the respective manuscripts in the system. The editorial management system however, does not only record which actor with which role releases or triggers an event. It also files who is affected by an event (Table 2). We have no insights into how triggering and affecting is defined for the infrastructure but can only infer from the fact that the infrastructure registers the person-ID as triggering or affected from its limited perspective. Again actors assigned editorial roles stand out, because their actions significantly affect actors with other roles assigned. For instance, 10,522 events triggered by editors affect referees. At the contrary, however, events triggered by authors and referees only affect events with actors assigned the same role. Thus, the heterogeneity of roles affected by editors shows their coordinating role in the process, due to what Reinhart and Schendzielorz have called the administrative practices of peer review.

**TABLE 2:** Events triggered by (columns) and affective to (rows) the different roles assigned.

	Author	Editor	None	Referee	Sum
Author	47,757	20	5	4	47,786
Editor	14	90,067	408	0	90,489
None	7	11,866	80,858	0	92,731
Referee	4	10,522	856	35,710	47,092
Sum	47,782	112,475	82,127	35,714	278,098

But there is a significant proportion of events triggered by actors with no role assigned (see Table 2). In any case, not assigning a role to some actors shows that those are regarded less relevant for the editorial process by design. Also, there are no actions recorded without two person-IDs involved, which means, that automated actions, if recorded, must be included with person-IDs. We therefore deduce, that the participant group of “none” roles must in part be comprised of non-humans (i.e., the infrastructure itself). A significant number of events (11,866, to be precise) released by editors affect actors with “none” specified roles. Given the administrative responsibilities of the editors, it is plausible that some of these events refer to quality or process control related activities such as setting up automated mailings without a call for action. If that assumption is right, administrative activities might indeed more closely be intertwined with what Schendzielorz and Reinhart (2020) have called observational activities (p.19), enlarging editor’s control on the process, but also putting more pressure on this role. Yet, given our limited reconstruction of the event history, we cannot confirm this hypothesis. More research would be needed in order to more closely reconstruct these events.

### Structure and Sequence of Events in the Process

**FIGURE 3:**
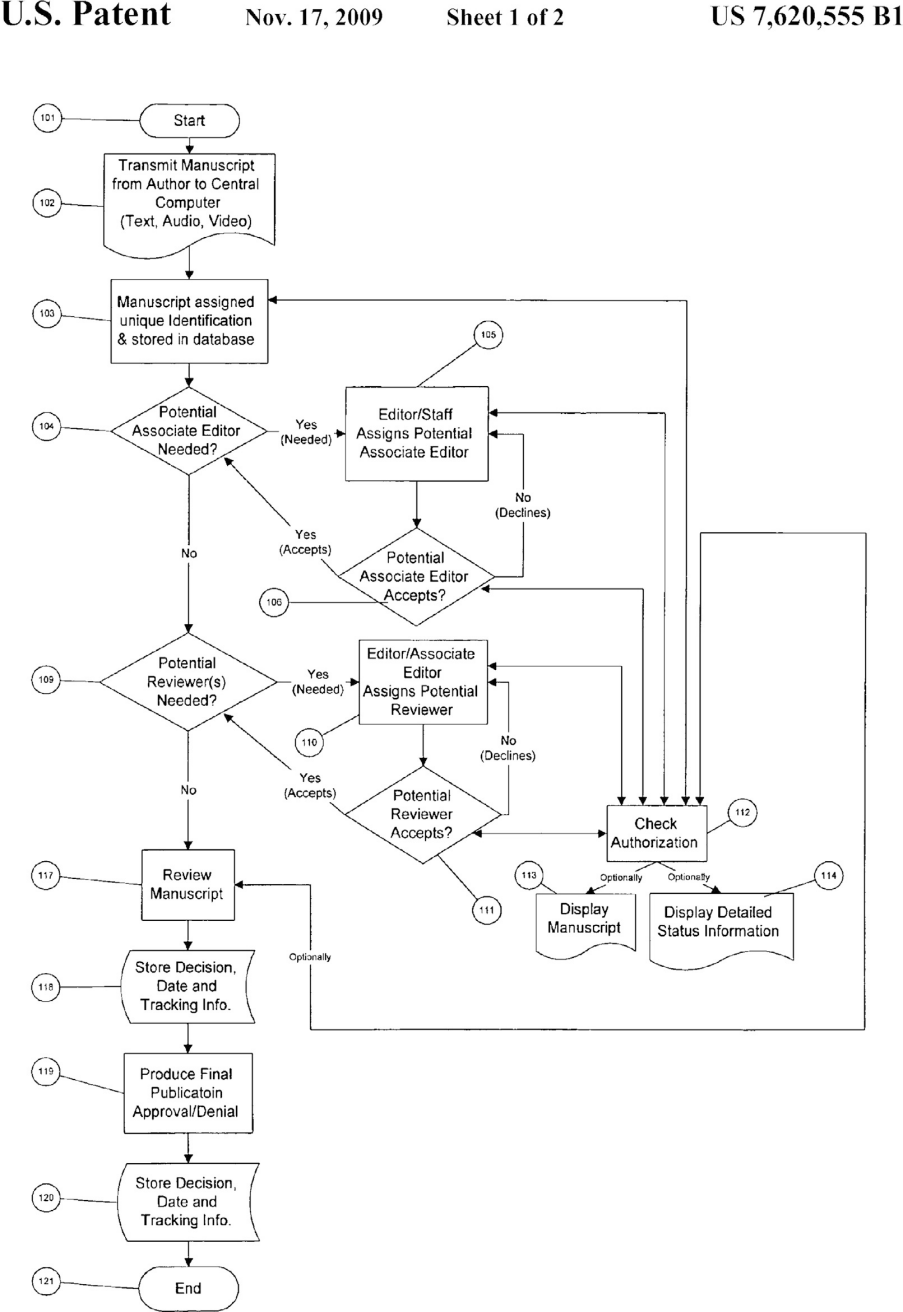
The editorial process as depicted in the patent (from: Plotkin (2009)).

In the patent’s process flow chart (see Figure 3), only 17 entities occur: start and end, six process items, four decisions, three documents and two storage operations. Also, there are only ∼29 directed links between the entities, resulting in a network density of ∼0.1, meaning that 10 percent of all theoretically possible edges occur. The patent shows the components like postulation, consultation and decision as elements relatively clearly, but the component of administration is distributed over the whole process. Also, it shows that there must exist parallel sub-processes (e.g., communication with different reviewers), which must, by construction, have been projected onto one timeline in the history dataset we were provided with. This dimensionality reduction probably obfuscates some properties of the implemented process, such as if it may have been acyclic in higher dimensionality, which we cannot observe any more, limiting the potential for our investigation.

**FIGURE 4:**
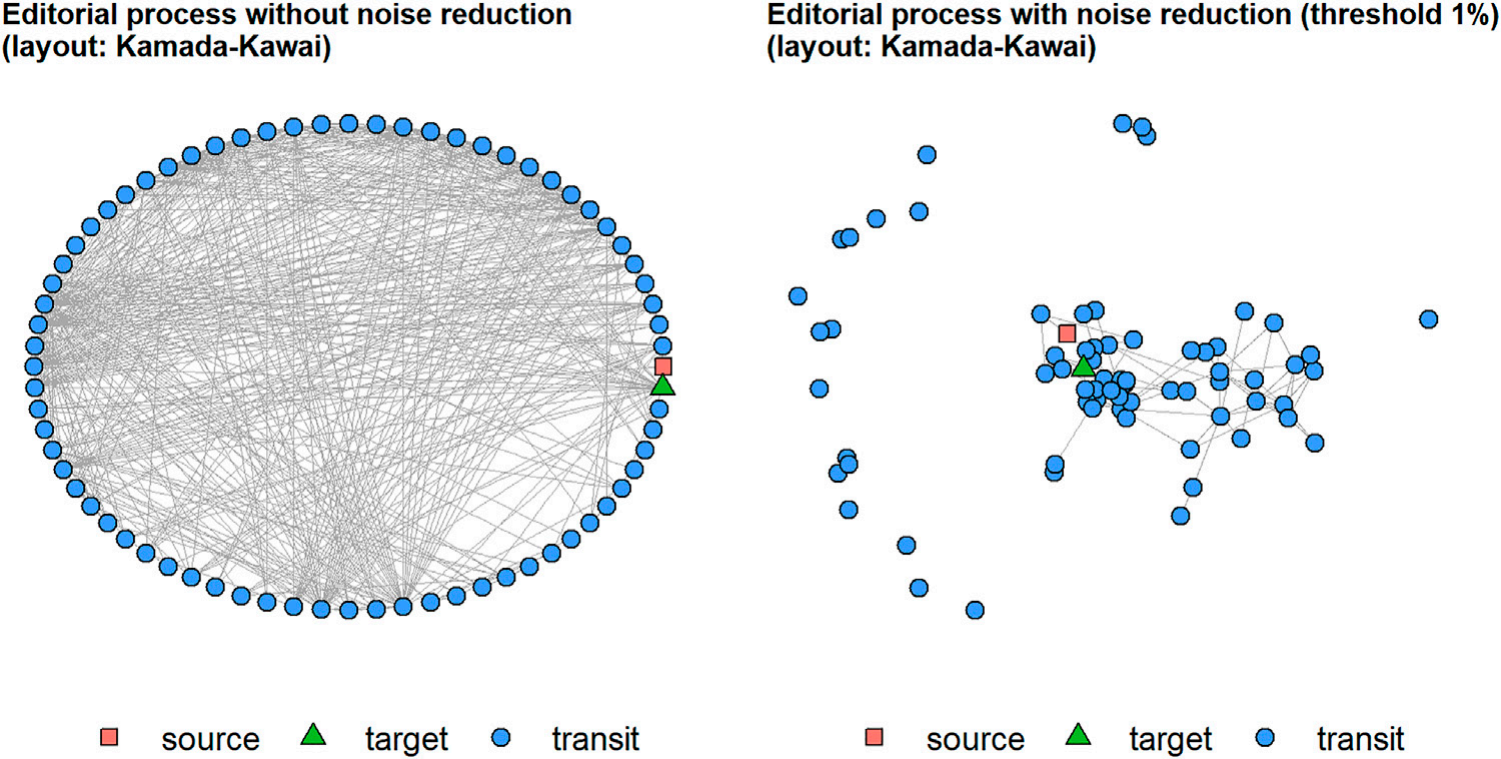
Order of the process without and with noise reduction. After noise-reduction, a core component emerges.

As described above, to investigate the idealized process from the patent empirically, we constructed a simplified network from the recorded events for all 14,391 first-version manuscripts, in which the nodes represent the stages and edges are drawn between two events which follow one another. The multiplicity of edges expresses how often its’ ends occur in direct sequence in the whole dataset, that means, for all first version manuscripts together. The two additional source and target nodes make start and end of the process visible. This network turned out to be relatively complex with 72 nodes and 623 edges, and relatively dense (with d = 0.12), which means, that 12 percent of all theoretically possible edges occur empirically. Because of combinatorial explosion, large networks can be expected to be less dense than smaller ones. Hence, a lower density in the observed network than in the patent would be more plausible for a streamlined process. However, in contrast to the patent for the editorial process, where steps have a clear order, the infrastructure seems to allow for an open process: in principle, almost any event could follow any other, which leaves the responsibility for the process in the domain of the actors. When we plot the network with Kamada-Kawai layout, the high network density causes the network to appear as a circle (see Figure 4, left) with no visually detectable pattern between source and target. Empirically, a panoply of orders occur in the manuscript histories, which means that for most of the stages, it is not predetermined in the system’s implementation what happens next in the process. If we rule out automated decision making (which we elaborate on later in this text). Hence, the infrastructure must offer its users a high degree of freedom regarding what they do next.

The process sequence is very open in principle, but for a process leading from submission to decision, some regularity in the steps could be expected, that is, some nodes must be more likely than others to be passed and also, some edges must be more important than others respectively. The given network cannot be completely chaotic, instead some structure must be there but need sharpening. So to reduce the noise and to uncover the core process, we deleted all edges, which had a multiplicity of less than 1% of the number of items. The remaining network has only 96 edges and a density of d = 0.02, and a core-periphery structure becomes visible (see Figure 4, right). We concentrate on the core process now and delete the now isolated vertices, thus reducing the core process to the main component of the network with 48 vertices and a density of d = 0.04.

To identify important passage points in the network, we chose node degree centrality with respect to edge multiplicity. Centrality is a relative measure, putting different nodes into an ordered relation. The most central node is “Preliminary Manuscript Data Submitted” which has 27,910 ingoing and outgoing edges, whereas the least central node is “Initial QC failed” (where QC stands for quality control) which has only 147 edges. As we were aiming at identifying core elements of the process, we disintegrate the graph into components by deleting the passage points in descending order by size to make its meaningful components fall apart from each other. We did not use a clustering algorithm, because those usually are based on cohesion or distance metrics: they regard those parts of graphs as different components, which are only weakly linked or distant from each other, whereas nodes belong to the same cluster component if they are strongly linked or close to each other. In contrast for our case, we hypothesize that the important things happen, where manuscripts differ from each other – this means that the passage points tend to carry less information about the process elements. For example, the event “Preliminary Manuscript Data submitted” happens for almost all manuscripts, which is why it does not help us to distinguish manuscript lifecycles in a meaningful way. On the other hand, “Initial QC failed” does not happen so very often and manuscripts facing this stage must have something special with them.

Accordingly, our process elements are strongly linked by the first couple of passage points, because they indicate states of transition. This led us to iteratively disintegrate the network by deleting the passage points. We stopped disintegration at the iteration before the four different decision events “Manuscript Rejected”, “Manuscript Revise and Re-Review”, “Manuscript Revise only” and “Manuscript Accepted” fell apart from each other into different components. Therefore we deleted the first nine passage points (including source and target). The disintegrated network consisted of eleven isolated components, of which 10 were consisting of three vertices or less and one component with 22 vertices, containing the decisions (see Supplementary Material).

**FIGURE 5:**
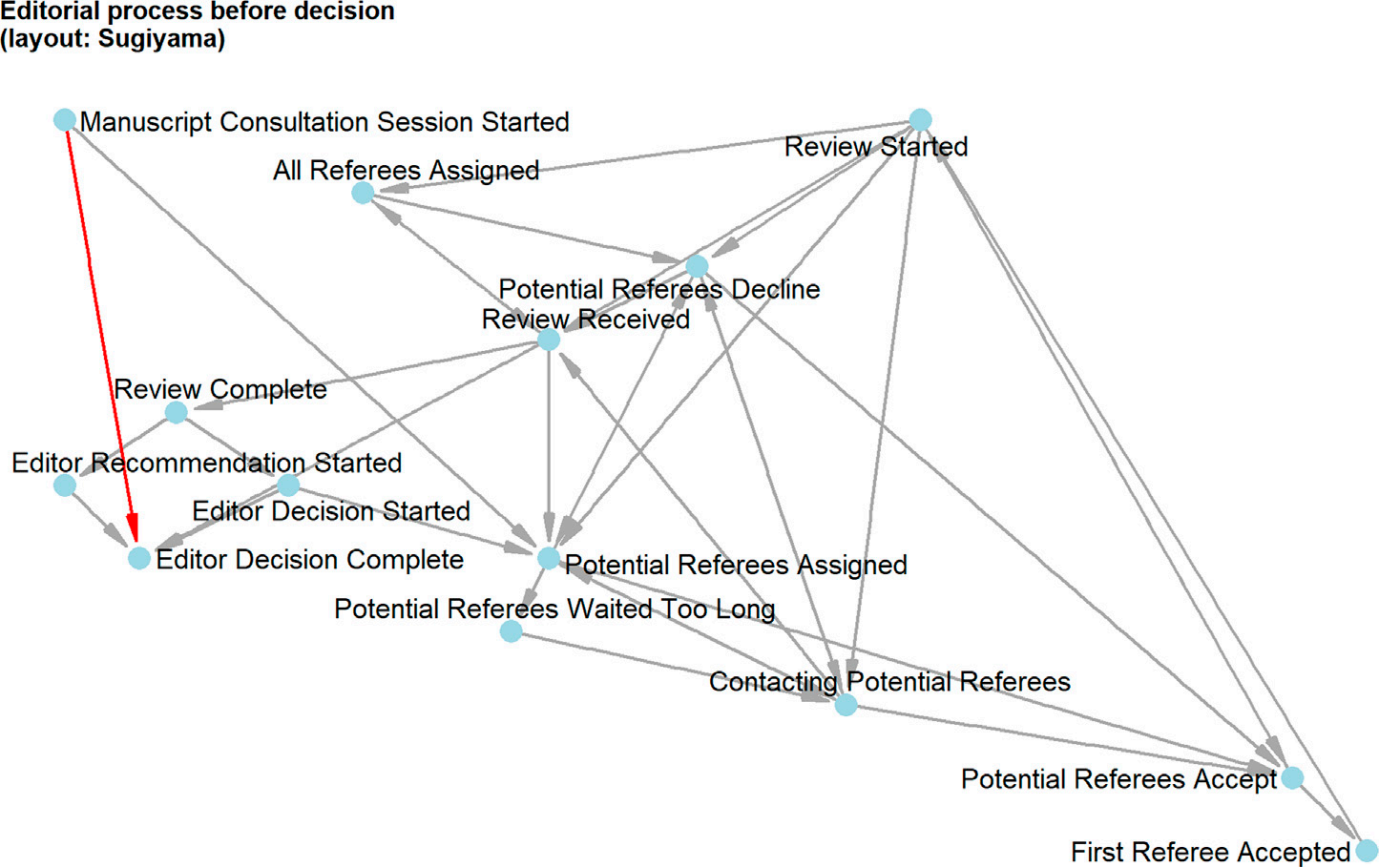
From the start of manuscript consultation until the editor’s decision: The figure shows that there is a short way (red) without external consultation and the long and complex way with external reviewers (grey).

**FIGURE 6:**
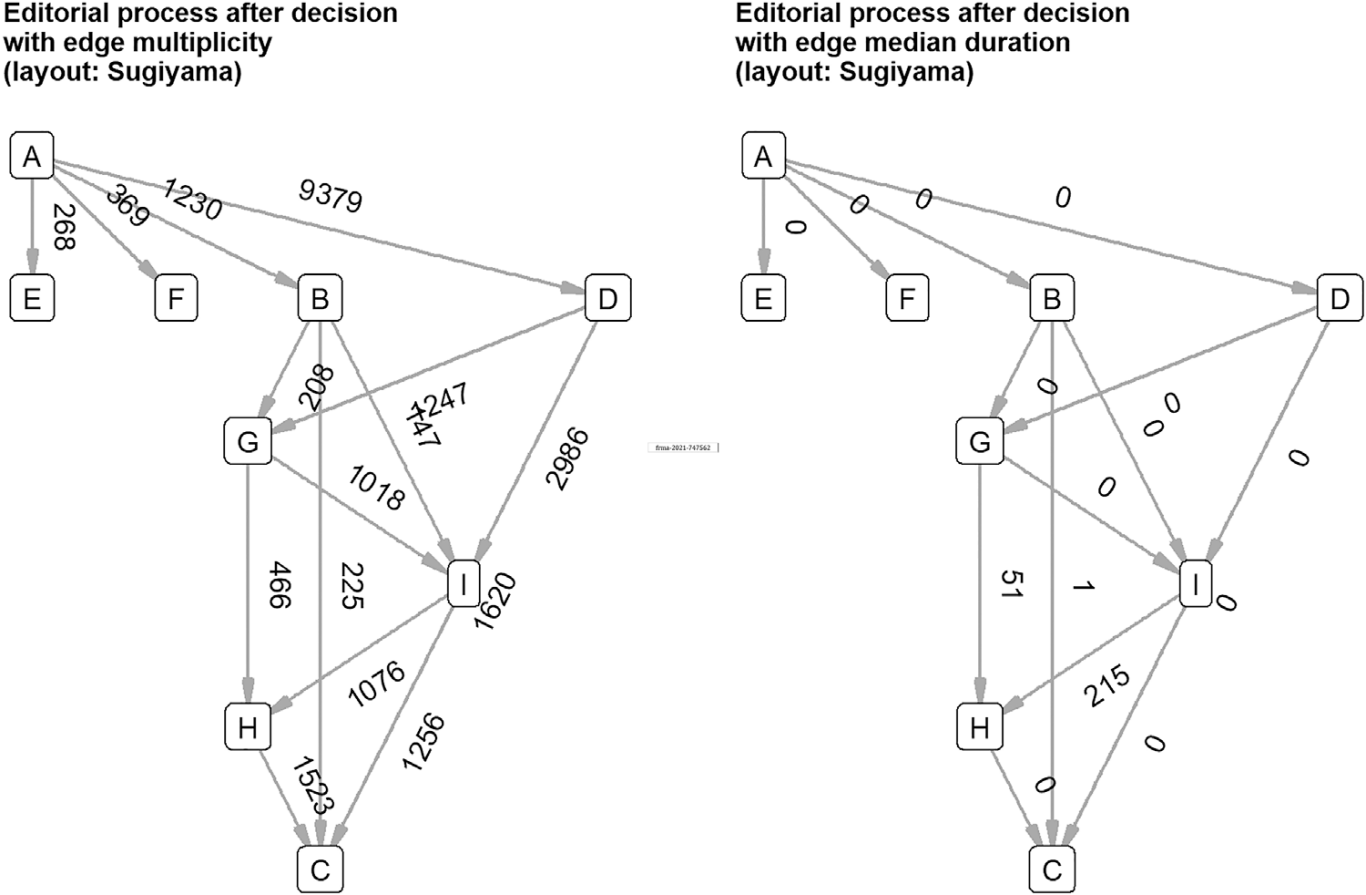
Events after decision with multiplicity and median duration show that editors thoroughly communicate about negative decisions. A—Editor Decision Complete, B—Manuscript Revise and Re-Review, C—Waiting to Send Decision to Author, D—Manuscript Rejected, E—Manuscript Revise Only, F—Manuscript Accepted, G—Drafting Decision Letter Started, H—Drafting Decision Letter Completed, I—Manuscript Consultation Session Ended.

The most interesting component of the disintegrated network was, of course, the one which included the four decision events. We found that there was a central vertex dividing the decision component in two parts: “Editor Decision Complete” is the demarcation between events before (review process) and after decision (decision communication). Before the decision, basically two things can happen (see Figure 5). The first possibility is the short decision path from “Manuscript Consultation Started” directly to “Editor Decision Complete”. The second possibility is the long decision path from “Manuscript Consultation Started” through external peer review to “Editor Decision Complete”. This matched with what we would have expected to happen: there are editorial decisions without peer review, which is also represented by the editorial management system. After the decision, four things can happen, but empirically, the four decisions can be divided into two groups (see Figure 6). The accepted manuscripts as well as those subject to revision are not processed further in this graph component. The rejected manuscripts and those to be resubmitted get a special treatment by the editors: the communication about the frustrating decision is thoroughly crafted showing in the network as two vertices about “Drafting Decision Letter”, notably resulting in longer durations for decisions to be sent to authors.

What is worth noting is that the content of reviewers’ opinions is not visible in the process, although the reviews are clearly processed by the infrastructure. We only find “Review Started” and “Review Received” in this respect, but we have, based on the event history only, no information as to what the reviewers might have recommended. From an ethnographic perspective this also means that the infrastructure itself cannot evaluate reviewers’ opinions due to its implementation and consequentially would not even be able to compile automated decisions. This underlines the strong position and great responsibility of the editor.

### Categorization of Events Identified

In order to get more insights which kinds of events are represented by the editorial management system inside the above mentioned core component with 48 nodes, and adapted by the publisher, we analysed their frequency for the whole dataset and tried to categorize them according to the heuristic provided by Schendzielorz and Reinhart. We did not categorize the source and target nodes as they were introduced throughout our analysis and not created by the system in the first place. We found that the labelling of the events indicates that at least all elements of the minimal model of peer review processes are represented, that is, postulation, consultation, administration and decision. The categorization table is attached as supplementary material to this paper.

Typically, events referring to what Schendzielorz and Reinhart (2020) have called postulation are triggered by the authors. We sorted seven events into this category (according to their labelling and the distribution of triggering roles), of which the event “Preliminary Manuscript Data Submitted” is the event with the highest frequency in the database (N = 16,901), followed by “Author Approved Converted Files” (N = 13,978). Also “Revision Received” (N = 2,498) was attributed to postulation representing a renewed claim of the author; and “Halted Manuscript Deleted” (N = 3,380) as this was triggered mainly by the authors. Furthermore, the following events were attributed to postulation: “Manuscript File Added” (N = 6,356), “Manuscript File Replaced” (N = 3,261) and “Manuscript Withdrawn” (N = 228), the latter being attributed to postulation because authors can decide as to whether they want to keep or withdraw their claim. These last three events were in the majority of the cases not recorded as triggered by the authors, but by the “none” role, displaying their additional observational or administrative character.

In the second category, which Schendzielorz and Reinhart (2020) have called consultation, we subsumed nine events, which are mainly performed by editors, reviewers and “none” roles. The editor decides about opening and closing the external review (expressed by “Manuscript Consultation Session Started” (N = 5,816) and “Manuscript Consultation Ended” (N = 2,010)). “Review Started” and “Potential Referees Accept” were mostly performed by the reviewer and achieved the highest frequency (both had N = 8,937). The identical numbers for both events indicate that they are released upon acceptance of the reviewer. The reviewers further triggered “Review Received” (N = 8,672), “First Referee Accepted” (N = 2,766) and “Review Complete” (N = 3,222), the latter indicating that a consultation event has actually taken place. Interestingly, when “Potential Referees Decline” (N = 7,743), this event is mostly triggered by a “none” role, because declining referees do not have a role with the manuscript in question. Also, “Editor Recommendation Started” (N = 431) was attributed to this category. Although, the latter sounds like a decision event, it is mainly recorded as triggered by the reviewers and is clearly located in the network before the decision.

Of major relevance for the peer review process is that it finally comes to a decision, based on consultation with internal and external actors. The preliminary analysis of events indicates that the editorial management system adapted in our case represents these activities with ample differentiation. Nine events could be attributed to this category, the most important being the four decision events “Manuscript Accepted” (N = 1,711), “Manuscript Revise Only” (893), “Manuscript Revise and Re-Review” (1,540) and “Manuscript Rejected” (9,835). The decision is framed by “Editor Decision Started” (N = 6,215, triggered often by the reviewer) and “Editor Decision Complete” (N = 13,973)—the difference in size indicates, that the editors’ decision can happen directly without external consultation. Surprisingly fine grained is the representation of the communication about the decision. While “Decision Sent to Author” plays a major role (N = 13,933), we also find a noteworthy amount of “Drafting Decision Letter Started” (N = 1,949) and “Drafting Decision Letter Completed” (N = 2,421). From an organizational perspective, the documentation of these events allows for carefully reconstructing and justifying difficult decisions, but it could also provide more insights into what happens at this stage of the process. In the subsection above, we have shown for first submitted versions that the drafting of decision letters happens mostly for negative decisions.

Nine events were attributed to the administrative activities of the peer review process, according to Schendzielorz and Reinhart (2020) comprising processes, where “postulations are received, their treatments are initiated or being coordinated”. The administrative procedures appear to be well covered by “Editor assigned” (N = 17,499), “Editor Replaced” (N = 561) and “Secondary Editor Replaced” (N = 333) as well as events indicating the contacting or assignment of reviewers: the editors choose the reviewers (expressed by “Potential Referees Assigned” (N = 10,888) and “Contacting Potential Referees” (N = 19,878)) and are informed about the outcome of their request with “All Referees Assigned” (N = 3,607). Also, “Manuscript Transferred” (N = 995), “Manuscript Ready for Publication” (N = 1,705) and “Manuscript Sent To Production” (N = 1,694) are events covering the transfer of publications after the review process was completed, referring to their relationship with the publishing house and their facilities.

Yet, in our data set, we also found events that reach beyond administrative activities, because they document pace, effectiveness, or quality of the process or the item (the manuscript), thus enabling quality control and supervision of the whole process, which we label “observational elements”. This category is comprised of “Waiting for Editor Assignment” (N = 14,261), “Waiting for Potential Referee Assignment” (N = 12,976), “Waiting to Send Decision to Author” (N = 5,796), “Waiting for Revision” (N = 2,612), “Waiting for Author Approval of Converted Files” (N = 898) and “Potential Referees Waited too Long” (N = 610). These events document the time passing before a relevant step in the consultation or postulation, inasmuch as they control if editors, authors and referees perform their tasks timely. Also, the initial quality control of manuscripts, indicated by the events “Initial QC Started” (N = 14,499), “Initial QC Complete” (14,288) and “Initial QC Failed” (N = 418) referring to the submission (where QC stands for quality control and the relation of failed versus complete initial quality controls shows that this event is mostly independent from the decision category), can be attributed to that category, because it potentially would also allow for detecting structural problems in the quality of submissions, thereby informing the controlling of the process. While the potential exploitation of these process generated data may support the administration, it at the same time may also put more pressure on the editor, simply because these data can be shared and discussed with potential stakeholders of the publisher.

Additionally, some events lie outside the categories of postulation, consultation, decision and administration as they indicate discussions. Apparently, appeal plays a minor role with “Waiting for Appeal” (N = 355), “Appeal Received” (N = 358) and “Appeal Request Accepted” (N = 355), but with overall low numbers.

## Discussion

We started our empirical analysis following the conceptual heuristics of Schendzielorz and Reinhart (2020), who provided elements of a minimal and maximum model of the peer review process. We are able to compare the elements and events described in the patent (Plotkin, 2009) with its adaptation at the publisher in question, where the elements of the process could only be identified by taking event labels, performing actors and sequence of steps together.

### The Editor Stays in Charge but is Supported by the Infrastructure.

We found that there is no standardized role for automated processing or decision making: the digital infrastructure itself is not explicitly listed as actor in the patent, but is only visible in the digital traces. In the patent, it says: “A user’s role includes one or more of the following relationships between the manuscript and the associated person: author, editor, associate editor, reviewer, or staff member.” (Plotkin, 2009 p.5). Furthermore, the editor is described as “optional” in the patent: “The publishing organization can, optionally, assign an editor, monitoring editor, or associate editor to oversee the review process […] and make the final publishing approval decision.” (Plotkin, 2009, p.4), but also the patent is open to an automated decision making. In the context of the editorial decision about publication, the inventors suggest: “Alternatively, the decision to publish may be automated based upon a ranking of the review decisions received from the reviewers.” (Plotkin, 2009, p.5). In contrast, in our data, the editors play a major role, performing lots of tasks affecting actors with other roles assigned and there is no automated decision making at play, when it comes to the final publishing approval decision. Also, the communication about the decision remains clearly in the editor’s hands, showing responsibility for the interaction with the scientific community.

The only aspect, for which we could not clearly reject the potential automated decision making was the “Initial Quality Control”—supposedly a check for a correctly completed submission form. This may show that the submission procedure is standardised, possibly making some forms of research impossible to submit. Also, the process as described in the patent and inscribed in the software would be technically open to integrate all kinds of checks at this point – even automated detection of content similarity with other papers as presupposition for plagiarism prevention. But instead, decision making and communication at the concrete journals under investigation clearly remain in the human domain. And, as the digital traces show, the editors carry them out thoroughly. This characteristic of the peer review process we must consider specific for this publisher, according to our data, and not a general feature, as the editorial management software could also be used otherwise.

With regard to roles and activities of the editor, there is support as well as control by the infrastructure. On the one hand, the observational procedures might help the editor to oversee whether other actors accomplish their tasks in time, on the other hand, actions of the editors are tracked as well. The performance of the editor can thus be controlled and evaluated by other stakeholders in the organization of the publisher.

Due to the specific work environment at the publisher, where editors are employed as full-time staff in a shared office space, it must be easy for them to communicate with each other bypassing the editorial management system, which limits the potential of surveillance through the system. Also, in contrast to what Taubert (2012) describes, we can assume, that the digital infrastructure in our case is not only imposed on the editors but is understood by them as a tool, which works – otherwise, they could adjust the system configuration or even collectively demand to abolish it. Additionally, due to the full-time character of the editorial work, a high proficiency with the system can be expected, which is confirmed by the fact that the process in practice is not so very much streamlined but the principal openness of the process order is occurring empirically in the data.

Authors as well as reviewers have no possibilities to bypass the system easily, as far as we can see. They can only choose to participate in it or not. The submission process is standardized through a web interface. In return, authors and reviewers experience less surveillance by the system, because only few formalized actions are recorded from them, because the system is clearly editor-centred.

### The Infrastructure Takes up the Administrative Perspective

**FIGURE 7:**
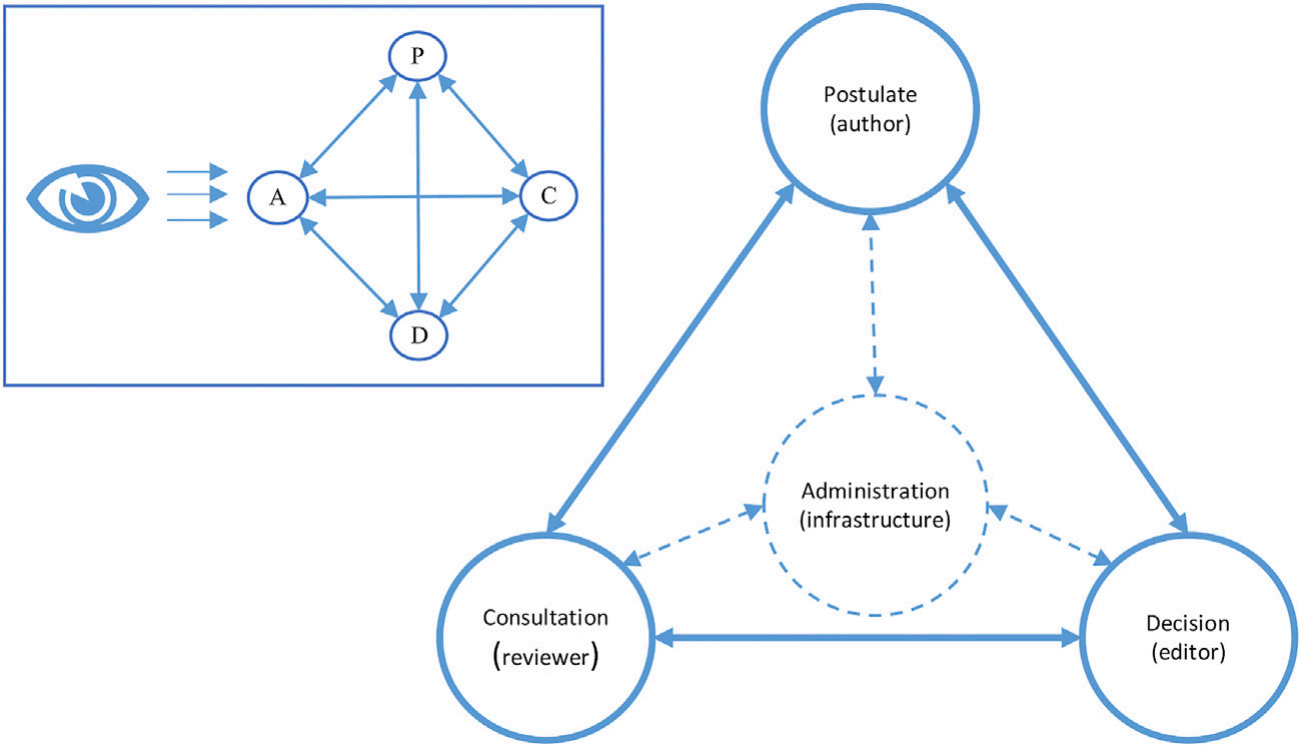
The process elements postulation (P), consultation (C), decision (D) and administration (A), adapted after Schendzielorz and Reinhart (2020), are mutually connected with each other, but seen by the infrastructure from the standpoint of administration. Thus, it is rendered invisible as distinguishable component.

The patent as well as the digital infrastructure aim at supporting the editor in their work. The patent shows a limited perspective on the peer review process, rendering the system itself invisible as a component (see Figure 7). In the minimal process of peer review according to Schendzielorz and Reinhart (2020), we would find the four processual elements being mutually connected with each other. In contrast, in the patent for our infrastructure, administration does not occur distinguishably in the process flow chart, but is distributed over the whole process making everything and nothing an administrative task. This is supported by the process sequence empirically showing regularities but being very open in principle. The editor-infrastructure compound – while overseeing the whole process – can only distinguish the other three components from each other, but cannot discriminate the administration.

Valuable insights were gained from the categorization of events into the process element categories. The strong presence of observational events underlines the property of editorial management systems being a knowledge based infrastructure enhancing the editor’s competence rather than only being a small tool. Consequently, we infer that the infrastructure becomes performative in the sense that an idealized model implemented as software defines what tasks are supported and which are neither supported nor tracked. On the other hand, the users of type editor seem to have much leeway regarding which tasks they choose to perform in which order, hence the empirical process network has so many different edges. This is exactly the reason why the digital infrastructure allows for the investigation of its users in so many different ways. In our case, the digital traces particularly point to the editors’ procedural choices.

The reviewers’ comments and recommendations are supposedly stored in the database at other places, but their content is not present in the manuscript histories – they only appear as “Review Received”. On the other hand, the editors’ decisions are stored in four different elements. This indicates, that administratively, the ongoing process is only indirectly affected by the reviewers’ recommendations, but directly affected by the editors’ decisions. This highlights the differences between the consultation and decision components of the process.

## Conclusion

We have shown in our contribution, that the peer review process in digital infrastructures is complex: We started from an abstract description of a minimal peer review process with four elements according to Schendzielorz and Reinhart (2020), acknowledged an ideal digitalized process with seventeen positioned components according to a patent (Plotkin, 2009) and empirically found an open process with 72 events in it. Also, we have found that participants in the process (see Schendzielorz and Reinhart, 2020) are translated into roles in the digitalized process (see Plotkin, 2009) and implemented as person-IDs in the digital infrastructure, only the latter distinctly displaying the infrastructure itself as an actor. The operationalization and implementation shows specific interpretations of the peer review process as an organizational activity. We preliminarily conclude that the partial perspective through the eyes of the digital infrastructure provides valuable insights into the peer review process, which are difficult to obtain otherwise.

We have also gained specific insights into how editors take their role in the peer review process seriously: despite automation of some administrative steps, decision-making as well as decision-communication remains in the human domain. Peer reviewers are assigned to manuscripts, reviewers’ recommendations are considered and the fate of a manuscript is decided about by the editor. Editors often communicate their decisions with individualised letters, putting much effort into decision-communication about non-successful submissions, which may show how they acknowledge authors’ individual pursuits of crafting and improving knowledge claims. Further, it indicates respect for the authors as sentient beings possibly frustrated about a negative decision. In the light of the transparent review process at this publisher, where editorial decision letters are published alongside accepted papers, this is especially interesting, because decision letters for successful submissions can be expected to have a much larger audience than for non-successful submissions. This may as well reflect how editors take their responsibility as members of the scientific community.

With respect to the tasks the editor performs, we can see that the editor is the most powerful actor in the process as represented in the traces of digital infrastructures as opposed to a more automated process powered by the infrastructure. Yet, the digital infrastructure accompanies each and every step of the editor, supporting the editor’s tasks, which could not be accomplished in an equal pace and magnitude without it. These representations on the one hand relate to the effort and the diversity of activities that go into scientific publishing (Taubert, 2016), but on the other hand, differences in the representation of peer review activities may also point to recent tensions in publishing as events indicating oversight or control may be expressions of commercial interest (Horbach and Halffman, 2019, p.12). Digital infrastructures, as Gillespie (2015) argued, are not neutral, but “intervene”. They enable, support or constrain some behaviours, but they can also make certain activities more visible and thereby more relevant than others, “they pick and choose” (ibid., 1). In this regard, editorial management systems perform timekeeping, when they support and oversee the duration of sub-processes (Reviewer Waited too Long, “Waiting for Authors Revision” etc.). By making these processes visible and measurable, the pace of the peer review process is reinforced as a relevant evaluation criterion for scholarly journals and their editors.

The study has several implications on the study of publishing practices and processes addressed in the article collection about “Change and Innovation in Manuscript Peer Review” it is part of. While focussing our analysis only on the case of one biomedical publisher, we may infer some more general observations for this realm of research. Digital infrastructures such as editorial management systems allow for processing data about the submission, evaluation and decision of manuscripts in novel ways, taking particularly the velocity, role specificity and consistence of the peer review process into account. Different to what may be expected by critical observers of digital platforms (Gillespie, 2015), editorial management systems do not always result in imposing pre-packaged models on scholarly publishing. Hence, there is no such thing as a uniform process put into place by a technology. As the case studied here shows, editorial management systems can be and are adapted to their context. The patented process is implemented as software, which is then adapted locally to the journal’s and publisher’s needs, taking stock of the diversity of scholarly publishing. This becomes particularly apparent when comparing the implemented structure observed with the patent published in 2009 showing an increase in complexity: while the patent is fixed in time, the software has evolved. At the same time, expectations that a stronger use of digital infrastructures would inevitably push forward innovations in peer review may be disappointed. There are certainly technological and organizational models in play fundamentally altering the role models of both reviewers and editors. In this specific case, however, the practices related to the technology support the principle of an editor centred system in the peer review process. This relates to recent research lines focusing on the stability and transformability of editorial practices by Horbach and Halffman (2020, p.3) arguing that existing editorial practices can be stabilized by infrastructures. Comparisons with novel digital infrastructures (and their implementations) for other publishers with different peer review models are necessary in order to more systematically judge or reflect on the influence of these infrastructural tools on innovation or stabilization in editorial work.

Yet, the analysis of processual data from an editorial management system may lead to research paying more attention to organizational issues of scholarly publishing, that is, practices related with maintaining and binding reviewers, authors and editors to a scholarly journal. These organizational and administrative practices may not always be related to epistemic values, yet they are an important part of scholarly knowledge production as scholarly journals are important sites for community building, safeguarding scientific quality and expectations to science in general. One issue for discussion in that process is the role of the editor. While the data explored do not allow for mining reviewers recommendations, and the data in this article say little about how editors deal with data about reviewers or authors, it does document well the various steps taken by the editors to reach to both authors and reviewers, to communicate and prepare selections and decisions. Consequently, the analysis shows how much organizational effort goes into what Schendzielorz and Reinhart (2020) have called the administrative parts of the peer review process to which this article pays particular attention. While these activities certainly would exist without editorial management systems, the latter makes them more visible and suspect to monitoring and optimization, because they can standardize editorial practices. As Horbach and Halffman (2020, p.4) have argued, such infrastructural “systems of classification and standards constitute ‘invisible mediators of action” establishing “templates (…) by which performances are compared and which define what one enactment is a performance of” (ibid).

## Data Availability

We were provided with data from an editorial management system by a biomedical publisher. We were allowed to analyse the data but not to share or publish the dataset. We store the data in our institute for 10 years according to the “Guidelines for Safeguarding Good Research Practice” (DOI: https://doi.org/10.5281/zenodo.3923602) by the German Research Association (DFG).

## References

[B2] Batagelj V. Ferligoj A. Squazzoni F. (2017). The Emergence of a Field: a Network Analysis of Research on Peer Review. Scientometrics 113, 503–532. 10.1007/s11192-017-2522-8 29056788 PMC5629241

[B3] Blümel C. (2021). “4.8 Academic Social Networks and Bibliometrics,” in Handbook Bibliometrics. Editor Ball R. (Berlin: De Gruyter), 255–264. 10.1515/9783110646610-026

[B4] Bös D. (1998). “Gedanken zum Refereesystem in ökonomischen wissenschaftlichen Zeitschriften,” in Von der Theorie zur Wirtschaftspolitik - ein Österreichischer Weg. Editors Baltzarek F. Butschek F. Tichy G. (Stuttgart: Lucius), 47–72.

[B5] Campanario J. M. (1998a). Peer Review for Journals as it Stands Today-Part 1. Sci. Commun. 19, 181–211. 10.1177/1075547098019003002

[B6] Campanario J. M. (1998b). Peer Review for Journals as it Stands Today-Part 2. Sci. Commun. 19, 277–306. 10.1177/1075547098019004002

[B7] Christin A. (2020). The Ethnographer and the Algorithm: beyond the Black Box. Theor. Soc. 49 (5), 897–918. 10.1007/s11186-020-09411-3

[B8] Cicchetti D. V. Rourke B. P. Wass P. (1992). Peer Review for Manuscript and grant Submissions: Relevance for Research in Clinical Neuropsychology. J. Clin. Exp. Neuropsychol. 14, 976–980. 10.1080/01688639208402548 1452641

[B9] Crane R. M. (1967). The Gatekeepers of Science: Some Factors Affecting the Selection of Articles for Scientific Journals. Am. Sociolog. 2, 195–201.

[B10] Csardi G. Nepusz T. (2006). The Igraph Software Package for Complex Network Research, InterJournal, Complex Systems 1695. Available at: https://igraph.org .

[B11] Csiszar A. (2018). The Scientific Journal: Authorship and the Politics of Knowledge in the Nineteenth century. Chicago: University of Chicago Press.

[B13] Dowle M. Srinivasan A. (2021). “data.table: Extension of `data.Frame`. R Package Version 1.14.0. Available at: https://CRAN.R-project.org/package=data.table .

[B14] Friedman B. Nissenbaum H. (1996). Bias in Computer Systems. ACM Trans. Inf. Syst. 14 (3), 330–347. 10.1145/230538.230561

[B15] Gillespie T. (2015). Platforms Intervene. Soc. Media Soc. 1, 1–2. 10.1177/2056305115580479

[B50] Glonti K. Boutron I. Moher D. Hren D. (2019). Journal Editor's Perspectives on the Roles and Tasks for Peer Reviewers in Biomedical Journals: A Qualitative Study. BMJ Open 9, e033421. 10.1136/bmjopen-2019-033421 PMC6886905 31767597

[B16] Grolemund G. Wickham H. (2011). Dates and Times Made Easy with Lubridate. J. Stat. Softw. 40 (3), 1–25. 10.18637/jss.v040.i03

[B17] Guston D. H. (2001). Between Politics and Science: Assuring the Integrity and Productivity of Research. Cambridge: Cambridge University Press.

[B18] Haraway D. (1988). Situated Knowledges: The Science Question in Feminism and the Privilege of Partial Perspective. Feminist Stud. 14 (3), 575–599. 10.2307/3178066

[B19] Harnad S. (1983). Peer Commentary on Peer Review: A Case Study in Scientific Quality Control. Cambridge: Cambridge University Press.

[B53] Hirschauer S. (2004). Peer Review Verfahren auf dem Prüfstand/Peer Review Research–Reviewed. Z. für Soziologie. 33, 62–83. 10.1515/zfsoz-2004-0104

[B20] Hirschauer S. (2010). Editorial Judgments. Soc. Stud. Sci. 40, 71–103. 10.1177/0306312709335405

[B23] Horbach S. P. J. M. Halffman W. (2019). Journal Peer Review and Editorial Evaluation: Cautious Innovator or Sleepy Giant? Minerva 58 (2), 139–161. 10.1007/s11024-019-09388-z

[B22] Horbach S. P. J. M. Halffman W. (2020). Innovating Editorial Practices: Academic Publishers at Work. Res. Integr. Peer Rev. 5, 11. 10.1186/s41073-020-00097-w 32774892 PMC7404921

[B49] Jubb M. (2015). Peer Review: The Current Landscape and Future Trends. Learn. Publish. 29, 13–21.

[B52] Kleimann B. Hückstädt M. (2001). Selection Criteria in Professorial Recruiting as Indicators of Institutional Similarity? A Comparison of German Universities and Universities of Applied Sciences. Qual. High. Educ.. 10.1080/13538322.2021.1889760

[B24] Krüger A. K. Hesselmann F. Hartstein J. (2021). “Bewertung in und durch digitale Infrastrukturen,” in Organisation und Bewertung. Editors Meier F. Peetz T. (Wiesbaden: VS Verlag für Sozialwissenschaften), 101–128. 10.1007/978-3-658-31549-8_5

[B25] Latour B. Woolgar S. (1979). Laboratory Life: The Construction of Scientific Facts. Beverly Hills: Sage Publishing.

[B26] Marres N. (2017). Digital Sociology: The Reinvention of Social Research. John Wiley & Sons.

[B27] Mendonça A. (2017). A Gestão On-Line Dos Manuscritos Na Profissionalização Dos Periódicos. Fisioter. Pesqui. 24 (2), 119. 10.1590/1809-2950/00000024022017

[B28] Mrowinski M. J. Fronczak A. Fronczak P. Nedic O. Ausloos M. (2016). Review Time in Peer Review: Quantitative Analysis and Modelling of Editorial Workflows. Scientometrics 107, 271–286. 10.1007/s11192-016-1871-z 27073291 PMC4819515

[B29] Niewöhner J. (2014). “Perspektiven der Infrastrukturforschung: care-full, relational, ko-laborativ,” in Schlüsselwerke der Science & Technology Studies. Editors Lengersdorf D. Wieser M. (Wiesbaden: Springer VS), 341–352. 10.1007/978-3-531-19455-4_28

[B30] Pedersen T. L. (2021). Ggraph: An Implementation of Grammar of Graphics for Graphs and Networks. R package version 2.0.5. Available at: https://CRAN.R-project.org/package=ggraph .

[B31] Plotkin J. F. (2009). U.S. Patent No. 7,620,555. Washington, DC: U.S. Patent and Trademark Office.

[B32] Pontille D. Torny D. (2015). From Manuscript Evaluation to Article Valuation: The Changing Technologies of Journal Peer Review. Hum. Stud. 38, 57–79. 10.1007/s10746-014-9335-z

[B34] R Core Team (2020). R: A Language and Environment for Statistical Computing. Vienna, Austria: R Foundation for Statistical Computing. Available at: https://www.R-project.org/ .

[B35] Reinhart M. (2010). Peer Review Practices: A Content Analysis of External Reviews in Science Funding. Res. Eval. 19 (5), 317–331. 10.3152/095820210x12809191250843

[B51] Röbbecke M. Simon D. (1999). Zwischen Reputation und Markt: Ziele, Verfahren und Instrumente von (Selbst)Evaluationen außeruniversitärer, öffentlicher Forschungseinrichtungen. (Papers / Wissenschaftszentrum Berlin für Sozialforschung, 99-002). Berlin: Wissenschaftszentrum Berlin für Sozialforschung gGmbH. https://nbn-resolving.org/urn:nbn:de:0168-ssoar-116609

[B37] Ross-Hellauer T. Deppe A. Schmidt B. (2017). Survey on Open Peer Review: Attitudes and Experience Amongst Editors, Authors and Reviewers. PLoS ONE 12, e0189311. 10.1371/journal.pone.0189311 29236721 PMC5728564

[B38] Schendzielorz C. Reinhart M. (2020). Die Regierung der Wissenschaft im Peer Review/Governing Science Through Peer Review. dms–der moderne staat–Zeitschrift für Public Pol. Recht Manage. 13 (1), 13–14. 10.3224/dms.v13i1.10

[B39] Seaver N. (2017). Algorithms as Culture: Some Tactics for the Ethnography of Algorithmic Systems. Big Data Soc. 4 (2), 2053951717738104. 10.1177/2053951717738104

[B40] Shapin S. (1994). A Social History of Truth. Chicago: Chicago University Press.

[B41] Star S. L. Bowker G. C. (2006). “How to Infrastructure,” in Handbook of New Media: Social Shaping and Social Consequences of ICTs. Editors Lievrouw L. A. Livingstone S. (London: SAGE).

[B42] Star S. L. (1999). The Ethnography of Infrastructure. Am. Behav. scientist 43 (3), 377–391. 10.1177/00027649921955326

[B43] Taubert N. (2012). Online Editorial Management-Systeme und die Produktion wissenschaftlicher Fachzeitschriften. Leviathan 40, 297–319. 10.5771/0340-0425-2012-2-297

[B44] Taubert N. (2016). “Open Access und Digitalisierung aus der Sicht von Wissenschaftsverlagen,” in Wissenschaftliches Publizieren: Zwischen Digitalisierung, Leistungsmessung, Ökonomisierung und medialer Beobachtung. Editors Weingart P. Taubert N. (Berlin: de Gruyter), 75–102.

[B45] van Noorden R. (2014). Online Collaboration: Scientists and the Social Network. Nature 512, 126–129. 10.1038/512126a 25119221

[B47] Weller A. C. (2001). Editorial Peer Review: Its Strengths and Weaknesses. Medford: Information Today.

[B48] Wickham H. Averick M. Bryan J. Chang W. McGowan L. François R. (2019). Welcome to the Tidyverse. Joss 4 (43), 1686. 10.21105/joss.01686

